# How Does Environmentally Specific Servant Leadership Fuel Employees’ Low-Carbon Behavior? The Role of Environmental Self-Accountability and Power Distance Orientation

**DOI:** 10.3390/ijerph19053025

**Published:** 2022-03-04

**Authors:** Yuhuan Xia, Yubo Liu, Changlin Han, Yang Gao, Yuanyuan Lan

**Affiliations:** 1 School of Economics and Management, Beijing Jiaotong University, Beijing 100044, China; 16113159@bjtu.edu.cn; 2 School of Business, Qingdao University, Qingdao 266071, China; hi1209@foxmail.com (Y.L.); hanchanglin00@163.com (C.H.); gyangfly@sina.com (Y.G.)

**Keywords:** carbon neutrality, environmentally specific servant leadership, environmental self-accountability, power distance orientation, low-carbon behavior, social learning theory

## Abstract

Environmental problems caused by excessive carbon emissions are becoming increasingly prominent and have received heightened attention in recent years. Encouraging people to adopt low-carbon behavior to reduce carbon emissions is desirable. Based on social learning theory, we developed and tested a moderated mediation model to investigate when and how environmentally specific servant (ESS) leadership impacts employees’ low-carbon behavior (i.e., private low-carbon behavior and public low-carbon behavior). We tested our theoretical framework with a sample of 483 subordinates and their direct supervisors working in northern China. The results indicate that ESS leadership is positively related to employees’ low-carbon behavior, and that environmental self-accountability plays a mediating role in this relationship. In addition, power distance orientation strengthens the direct effects of ESS leadership on employees’ environmental self-accountability and low-carbon behavior, as well as the indirect effect of ESS leadership on private low-carbon behavior via environmental self-accountability. Our findings contribute to the literature surrounding ESS leadership and low-carbon behavior, and help to promote green development and thus achieve the goals of carbon neutrality and decreasing carbon dioxide emissions.

## 1. Introduction

The observed increases in carbon dioxide concentration has led to numerous serious environmental problems, such as climate change, sea-level rise, and species extinction [1,2]. Such problems have received increasing attention in recent years. In order to address these issues, UN2030 Agenda, Cop26, and Fit for 55 have called for a reduction in carbon emissions. Specially, the Chinese government has decided to adopt more effective measures to realize peak carbon dioxide emissions by 2030, and strive to achieve carbon neutrality by 2060. It is worth noting that the increase in carbon emissions is related to industrial development and individuals’ daily behavior. Research shows that carbon emissions in individuals’ daily lives account for 80% of the global total carbon emission [3]. Therefore, given the massive scale of carbon emissions and the continuous accumulation of environmental pressure, it is necessary to improve individuals’ low-carbon awareness and boost individuals’ low-carbon behavior through their daily life choices [4,5,6]. Low-carbon behavior includes using energy-saving appliances, turning off appliances when they are not in use, as well as other actions that help build a low-carbon society [7]. Given that the behavior of individuals is more susceptible to influence by those with higher organizational status, such as leaders [8,9], it is necessary to discuss the impact of leadership on the low-carbon behavior of employees. Environmentally specific servant (ESS) leadership is defined as leadership that prioritizes environmental interests ahead of personal and corporate interests, with a focus on building pro-environmental values among organizational stakeholders [10]. By focusing on the needs of subordinates, ESS leadership strives to inspire environmentally friendly values among stakeholders (including employees and customers) to maintain the community [10], and thus may shape subordinates’ low-carbon behavior. Therefore, it is necessary to explore the relationship between ESS leadership and low-carbon behavior. However, it is unclear whether ESS leadership significantly affects low-carbon behavior, and how and when this effect occurs.

Previous research on low-carbon behavior focuses predominantly on government, region, and corporate levels [11,12,13]. For example, Du and colleagues [14] investigated how carbon tax impacts the low-carbon behaviors of construction stakeholders. Zhang et al. [15] surveyed Hangzhou, China, at the regional level to specify the impact of residential self-selection on low-carbon behavior. Moreover, at the corporate level, Sun et al. [16] analyzed the relationship between low-carbon behavior, economic transformation, and financial performance in listed companies in China. Up to now, research into low-carbon behavior at an individual level is scarce. Furthermore, most of this research has concentrated on low-carbon consumption behavior [17]. Liu and colleagues [18], for example, looked into the factors that influence college students’ low-carbon consumption habits, and Yin and Shi’s [19] research found that social interaction is an essential predictor of residents’ low-carbon consumption habits. Although research into low-carbon behavior is still emerging, and previous research has shown that leadership styles (i.e., eco-centric leadership, spiritual leadership) significantly influence followers’ environmental behaviors [20,21], few studies have examined the influence of ESS leadership on subordinates’ low-carbon behaviors. In addition, the existing research objects of low-carbon behavior are mostly residents and consumers [22,23]. Research that examines low-carbon behavior in the context of employees who work for an organization is limited.

In order to address the above issues, the current research aims to explore the impact of ESS leadership on low-carbon behavior based on social learning theory. Social learning theory proposes that individuals learn by observing and imitating the attitudes, values, and behaviors of role models in society [24]. Leaders are a crucial source of learning for subordinates in the workplace, especially when leaders are regarded as role models [24,25]. In their work, Hunter and colleagues [26] further pointed out that leaders are a vital source of role modeling due to their status and power over followers, especially when they are perceived as credible role models. Hence, ESS leadership may give rise to employees’ low-carbon behavior.

**Figure 1:**
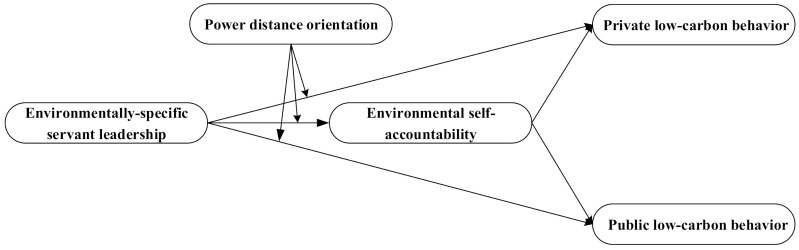
Conceptual model.

Moreover, ESS leaders’ attitude towards the environment in their work may be observed and learned by their subordinates. Consequently, subordinates’ eagerness to protect the environment according to internal self-standards is stimulated [27]. Such experience is combined with an increase in environmental self-accountability. As a result of the awakening of environmental self-accountability, employees are predicted to engage in low-carbon behavior more actively. Thus, this research attempt to explore the specific mechanism—environmental self-accountability—that links ESS leadership to employees’ low-carbon behavior. In addition, per social learning theory, the effect of social learning varies with different learners [28]. It is notable that, compared with Westerners, Chinese people are deeply influenced by Confucian culture and usually have a high power distance orientation. Although China is a country with a high level of power distance, the extent to which individuals accept unequal distribution of power in institutions and organizations is different [29]. Individuals with higher power distance orientation respect and rely more on their leaders; thus, they are more susceptible to leaders’ influence. Therefore, this research takes power distance orientation as a moderator to investigate the different effects of ESS leadership on low-carbon behavior for employees with different cultural values. Figure 1 shows the conceptual model.

This research contributes to the existing literature in three ways. First, the relationship between ESS leadership and low-carbon behavior is not well understood because no empirical research has tested this possible relationship. Understanding the impact of ESS leadership helps obtain a more complete picture of how ESS leadership works. Second, although previous research explored possible boundary conditions of ESS leadership (i.e., perceived organizational support) [30], the effects of subordinates’ cultural values are overlooked. Exploring power distance orientation as an essential condition factor of ESS leadership’s influence on low-carbon behavior provides a novel understanding of the limitations of ESS leadership. Third, this research complements empirical research by uncovering the mediating role of environmental self-accountability, bridging the broader literature on leadership and behavior.

## 2. Theory and Hypotheses

To investigate the relationship between ESS leadership and low-carbon behavior, we draw upon social learning theory, which explains human behavior from the perspective of the “continuous reciprocal interaction between cognitive, behavioral, and environmental determinants” [28]. A key tenet of social learning theory is that individuals learn behavior “through modeling: from observing others one forms an idea of how new behaviors are performed, and on later occasions, this coded information serves as a guide for action” [28]. Social learning theory argues that there are four stages for the processes of observational learning. First, observers must pay attention to the features of the modeled behavior; second, observers must represent the modeled behavior in memory; third, observers need to covert the modeled behavior into their own actions; finally, the last stage is the motivational processes, which determines whether there are matching responses between observers’ behavior and modeled behavior [28]. Moreover, social learning theory indicates that an individual’s behavior is shaped by environmental factors, cognitive factors, and the outcomes of their own behaviors. Observational learning is not always equally effective; it depends on the features of models, observer’s traits, and the outcomes of the matching behaviors [28].

ESS leadership promotes environmental values, attitudes, and actions in the workplace. Following the logic of social learning theory, by considering the ESS leader as a role model, followers are more likely to gain a sense of high environmental self-accountability and participate in more low-carbon behavior.

### 2.1. Environmentally Specific Servant Leadership and Low-Carbon Behavior

Many scholars have recently paid attention to environmental protection [31], leading to the development of the new concept of ESS leadership. Compared to servant leadership, ESS leadership emphasizes promoting stakeholders’ pro-environment behaviors [32]. Specifically, as environmentally oriented servant leadership, ESS leadership grants guidance to and incentivizes subordinates to be pro-environment citizens and demonstrates their stewardship and authenticity in creating a sustainable society [10]. In order to achieve the above goal, ESS leaders adhere to the belief of “people first”, stimulating employees’ green awareness and fostering them to establish environmentally friendly values by teaching them knowledge and skills related to environmental protection [10,33,34].

Similar to pro-environment behavior, low-carbon behavior is mentioned to describe the behavior of establishing a low-carbon society through low-carbon consumption, low-carbon electricity saving, and other behaviors to reduce energy consumption [7]. Stern [35] divided low-carbon behavior into two aspects. The first is private low-carbon behavior (e.g., purchasing, using, and disposing of personal and household products or services that impact the environment). The second is public low-carbon behavior, which indirectly impacts the environment by influencing public policy or others’ environmental behavior (e.g., petitioning environmental issues, supporting environmental policies, and encouraging others to participate in environmental activities). Low-carbon behavior at the personal level plays a crucial role in any potential shift to a low-carbon society [7]. In addition to reducing direct emissions (e.g., saving gas or electricity at home), individuals play multiple roles in contributing to a low-carbon society [36], including becoming low-carbon consumers [37], low-carbon employees [37], and low-carbon citizens [38]. In terms of public low-carbon behaviors, according to Stern [35], such behaviors can further indirectly affect government decisions by influencing the social environment, thus enabling a broader population to engage in low-carbon behaviors.

This research argues that ESS leadership positively influences low-carbon behavior. To begin with, in conjunction with social learning theory, individuals can acquire new behavior patterns by observing the behavior of others in a social system and imitating the behavior of essential role models around them [28]. Thus, employees are inclined to imitate leaders in the workplace when they are regarded as reliable role models [39]. As mentioned above, the features displayed by servant leaders (e.g., no reward required, putting employees first, and loyalty) will be identified by employees and promote the leaders to an object of emulation [40]. As a kind of servant leader, ESS leaders practice what they preach and act to protect the environment [10]. Therefore, employees would follow and imitate ESS leaders in consciously saving energy and resources and encourage their family members and friends to do the same.

Moreover, since ESS leaders are devoted to green goals, they can provide employees with environmental protection knowledge and skills to encourage them to conduct environmental behaviors [32]. Existing empirical research supported our speculation and argued that ESS leadership could ramp up employees’ green behavior by creating a green climate and changing their green actions [32,41]. Accordingly, this research hypothesizes that:

**Hypothesis** **1.** *Environmentally specific servant leadership is positively related to (a) private low-carbon behavior and (b) public low-carbon behavior.*

### 2.2. The Mediating Role of Environmental Self-Accountability

Environmental self-accountability is defined as the desire of individuals to live up to their environmental self-standards [42]. This concept of environmental self-accountability came from the relevant research on self-responsibility in marketing. Individuals with high environmental self-accountability tend to consume environmentally according to ethical and sustainability standards [43,44]. Besides, employees with high environmental self-accountability adhere to environmental and behavioral norms and evaluate or adjust their behavior based on these criteria [42]. In other words, environmental self-accountability is likely to stimulate employees to justify their green actions to self-identity or self-image shaped by their beliefs, values, or standards [45,46,47]. Thus, employees with high environmental self-accountability would show more altruism and responsibility and regard environmental protection as the guiding principle.

Drawing on social learning theory, this research speculates that ESS leadership positively influences employees’ environmental self-accountability for the following reasons. First, Bandura [28] indicated that individuals tend to observe and imitate the behaviors, attitudes, and values of role models who are important in their environment and acquire new behavior patterns through observational learning. As mentioned above, leaders who possess excellent ESS leadership traits will be considered role models and become an object of emulation [48]. Such imitation will be embodied in followers’ outwardly observable behaviors and reflect their values of vigorously protecting the environment [8]. Consequently, employees are likely to learn the responsibility and green awareness toward the environment shown by the ESS leaders, increasing environmental self-accountability. Second, ESS leaders inculcate self-sacrificing behavior in their employees for the greater good of society, e.g., protecting the environment [49]. Hence, ESS leadership could foster environmental self-accountability among employees by internalizing green values [50]. Therefore, employees could develop a high level of environmental self-responsibility and practice the green mission consciously and autonomously. This is consistent with the research of Jiang and colleagues [51], which suggests that environmental leadership can effectively shape followers’ environmental valves. Thus, we argue that ESS leadership could enhance employees’ environmental self-accountability.

This research argues that ESS leadership can improve employees’ environmental self-accountability and assumes that environmental self-accountability is positively related to employees’ low-carbon behavior. Bandura [24], Bower [52], and Neisser [53] deemed that expectations, self-perceptions, beliefs, intentions, and goals give direction and shape to behavior. That is, what people think, believe, and feel affects how they behave. As a kind of self-responsibility, environmental self-accountability is the willingness to practice standards of behavior and the prerequisite for individuals to choose their behavior [54]. This makes it a critical cognitive–psychological mechanism that drives employees’ low-carbon behavior. Thus, employees are more likely to adhere to behavioral norms resulting from environmental self-accountability and to assess or adjust their behavior based on these standards [42]. Consequently, there is a potential for a marked increase in employees’ low-carbon behavior. 

Following the above analysis, this research goes a step further and posits that environmental self-responsibility plays an essential mediating role in the leadership–employee behavior path. Under the guidance of leaders, employees classify themselves as socially and environmentally responsible citizens by improving self-accountability, which consequently spurts them to engage in more low-carbon behavior. Specifically, they tend to promote environmentally friendly living, practice private low-carbon behavior, and provide a path that indirectly influences public policy and other environmental behavior. Therefore, this research hypothesizes that:

**Hypothesis** **2.** *Environmental self-accountability mediates the relationship between environmentally specific servant leadership and (a) private low-carbon behavior and (b) public low-carbon behavior.*

### 2.3. The Moderating Role of Power Distance Orientation

Power distance orientation refers to how individuals accept unequal power distribution in institutions and organizations [55]. The acceptance of inequalities in power predicts how individuals interact with different levels of power [56]. For example, employees with higher power distance orientation are more aware of differences in status during the interaction process. They are more likely to obey the decisions of their superiors (e.g., even wrong decisions). On the contrary, employees with lower power distance orientation care more about the equal relationship with the leaders and are less grateful to the leaders [57].

Through the lens of social learning theory, the influence of the demonstrator on the observer is affected by the differences in observers [28]. Aligning with this idea, this research speculates that power distance orientation positively moderates the impact of ESS leadership on employees’ low-carbon behavior. As previously mentioned, individuals higher in power distance orientation are more likely to respect, defer to, and trust an authority [29]. Employees with high power distance orientation are more eager to implement the environmental protection measures ordered by ESS leaders. Furthermore, they are more likely to imitate ESS leaders’ acts performed for environmental protection and are more willing to apply knowledge learned from leaders in daily life to conduct private low-carbon behavior than employees with low power distance orientation [32,39,58]. Moreover, employees with higher power distance orientation are more willing to obey authority figures. They have more trust and recognition with leaders [59]. Thus, high power distance orientation associates are more likely to internalize the environmentally friendly nature of ESS leadership, which drives them to focus on the environmental problems and bring those around them to join in the environmental activities to fulfill public low-carbon behavior.

Power distance orientation affects individuals’ behavior and significantly impacts their thinking [54,60]. Thus, this research supposes that power distance orientation moderates the relationship between ESS leadership and low-carbon behavior and between ESS leadership and employees’ environmental self-accountability. As mentioned earlier, employees with high power distance orientation show more respect and trust in authority. They are autonomous in learning the values that ESS leaders uphold [29]. Thus, when ESS leaders demonstrate the values and attitude associated with protecting the environment in their work, high power distance orientation individuals are more willing to emulate this value and show more environmental self-responsibility. On the contrary, employees with low power distance orientation are more likely to view leaders as equals and not submit to their authority [57]. Therefore, leadership values are not worth learning for low power distance orientation employees. Furthermore, they prefer to stick to their values compared with those who regard the leader as a model. This concludes that employees with low power distance orientation are less motivated to learn from ESS leaders than employees with high power distance orientation. Thus, this research hypothesizes that:

**Hypothesis** **3.** *Power distance orientation moderates the positive relationship between environmentally specific servant leadership and employees’ (a) private low-carbon behavior and (b) public low-carbon behavior, such that these relationships will be stronger (i.e., more positive) for employees reporting higher as opposed to lower power distance orientation.*

**Hypothesis** **4.** *Power distance orientation moderates the positive relationship between environmentally specific servant leadership and employees’ environmental self-accountability, such that this relationship will be stronger (i.e., more positive) for employees reporting higher as opposed to lower power distance orientation.*

According to social learning theory, individuals’ characteristics affect the acceptance and absorption of indirect experience gained from other aspects. Logically, employees with high power distance orientation are more likely to be influenced by ESS leadership. They are more inclined to recognize the status gap between themselves and their superiors and agree with ESS leaders’ green values, reflecting more private low-carbon behavior. Additionally, there is a preference for employees with high power distance orientation to engage in public low-carbon behavior by persuading team members, friends, and family members to comply with green norms and encouraging them to participate in the activities. Accordingly, this research hypothesizes that:

**Hypothesis** **5.** *Power distance orientation moderates the indirect effect of environmentally specific servant leadership on employees’ (a) private low-carbon behavior and (b) public low-carbon behavior via environmental self-accountability, such that these indirect effects will be stronger for employees reporting higher as opposed to lower power distance orientation.*

## 3. Methods

### 3.1. Sample and Procedure

Our data came from several companies located in Shandong Province, China. The industries included textiles, food processing, and battery, which consume large amounts of energy and electricity and emit large amounts of carbon dioxide. With the help of those companies’ human resources management departments, a total of 650 subordinates and 216 immediate supervisors volunteered to participate in our survey. All participants were presented a 15-min orientation meeting in their own company. In this meeting, we explained the purpose of this survey, emphasizing the importance of how the participants think and feel. We also guaranteed that all their responses would be confidential and anonymous. Unique identification codes were assigned to each participant to match supervisor–subordinate responses. Respondents were required to write down their codes before submitting the questionnaire. Moreover, each completed questionnaire was placed in a separate sealed envelope. Participants received ten yuan in exchange for completing the questionnaire.

To reduce the detrimental effects of common method bias, we not only used the supervisor–subordinate dyadic design, but also adopted a multi-wave design with a one-month interval. At time 1, subordinates rated ESS leadership, power distance orientation, and covariates (i.e., demographic details and environmentally specific transformational leadership); 603 completed questionnaires were collected at time 1. One month later, at time 2, subordinates were asked to rate their environmental self-accountability, and 524 completed questionnaires were collected. One month later at time 3, supervisors rated subordinates’ low-carbon behavior, and 182 completed supervisor questionnaires were collected.

To improve data quality, we took further steps to remove invalid responses. For example, if the time taken to complete the questionnaire was less than half the average time needed for questionnaire completion, or the same choice was selected in the whole questionnaire, or the wrong choice was selected for the attention check items. Subsequently, we matched the completed supervisor and subordinate questionnaires. Finally, the valid samples comprised 483 subordinates and 182 supervisors, yielding a 74.31% response rate of subordinates and an 84.26% response rate of supervisors. The average age of the 483 subordinates was 30.99 years old (*SD* = 7.27); 60.04% of the subordinates were male; and 48.24% held an undergraduate degree or higher.

### 3.2. Measures

We translated all the English scales into Mandarin Chinese following Brislin’s [61] back-translation procedure. Unless mentioned otherwise, all items were rated on a 7-point Likert scale from 1 = strongly disagree to 7 = strongly agree.

Environmentally specific servant leadership. Subordinates rated their supervisors’ environmentally specific servant leader behaviors with a 12-item scale adapted by Luu [30] from Liden et al. [62]. A sample item is “My leader emphasizes the importance of contributing to the environmental improvement” (Cronbach’s α = 0.911).

Environmental self-accountability. Subordinates reported their environmental self-accountability with a 3-item scale developed by Peloza et al. [42]. A sample item is “I feel accountable for my own environmental self-standard” (Cronbach’s α = 0.754). 

Low-carbon behavior. Supervisors were asked to rate their subordinates’ low-carbon behavior with a 9-item scale developed by Bai and Liu [38]. Low-carbon behavior is composed of private low-carbon behavior and public low-carbon behavior. A sample item for private low-carbon behavior is “This subordinate saves energy and resources” (Cronbach’s α = 0.754). A sample item for public low-carbon behavior is “This subordinate supports low-carbon policies” (Cronbach’s α = 0.806).

Power distance orientation. We used a 6-item scale adapted by Dorfman and Howell [63] to measure subordinates’ power distance orientation. A sample item is “Managers should make most decisions without consulting subordinates” (Cronbach’s α = 0.859).

Control variables. Consistent with prior research [64], we controlled participants’ age, gender, and education level. Given that other leadership styles may also affect low-carbon behavior, we controlled environmentally specific transformational leadership. Subordinates rated their supervisors’ environmentally specific transformational leadership with a 12-item scale developed by Robertson [65]. A sample item is “My leader acts as an environmental role model” (Cronbach’s α = 0.922).

## 4. Results

### 4.1. Confirmatory Factor Analysis

To examine the discriminant validity of the focal construct in this research, a confirmatory factor analysis was conducted with Amos 23. As shown in Table 1, the six-factor model (i.e., environmentally specific transformational leadership, power distance orientation, ESS leadership, environmental self-accountability, private low-carbon behavior, and public low-carbon behavior; χ^2^ = 952.287, df = 804, χ^2^/df = 1.184, CFI = 0.983, TLI = 0.981, RMSEA = 0.020, SRMR = 0.035) displays a better fit than other five models.

### 4.2. Descriptive Statistics

Means, standard deviations, and correlations are shown in Table 2. The results suggest that ESS leadership is significantly positively related to environmental self-accountability (r = 0.331, *p* < 0.01), private low-carbon behavior (r = 0.299, *p* < 0.01), and public low-carbon behavior (r = 0.296, *p* < 0.01). Environmental self-accountability is significantly positively related to private low-carbon behavior (r = 0.391, *p* < 0.01) and public low-carbon behavior (r = 0.311, *p* < 0.01).

### 4.3. Hypotheses Testing

To test hypotheses 1 to 5, this research constructed a structural equation model using maximum likelihood estimation along with 5000 bootstrap estimations. As shown in Table 3, ESS leadership is positively associated with private low-carbon behavior (β = 0.163, 95% CI = [0.095, 0.232]) and public low-carbon behavior (β = 0.163, 95% CI = [0.095, 0.232]), supporting Hypothesis 1. The results in Table 3 also indicate that environmental self-accountability plays a significant mediating role in the relationship between ESS leadership and low-carbon behavior. For private low-carbon behavior, the indirect effect is 0.084 (95% CI = [0.049, 0.119]); for public low-carbon behavior, the indirect effect is 0.052 (95% CI = [0.023, 0.082]), supporting Hypothesis 2.

**Figure 2:**
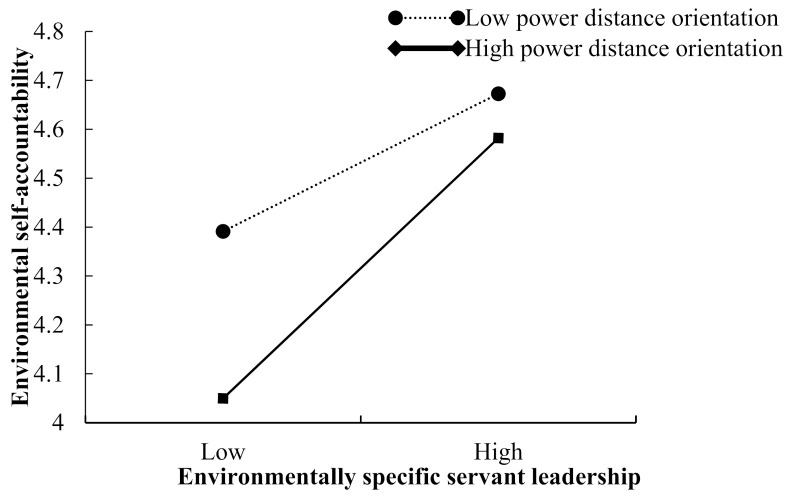
The moderating effect of power distance orientation on the relationship between environmentally specific servant leadership and environmental self-accountability.

**Figure 3:**
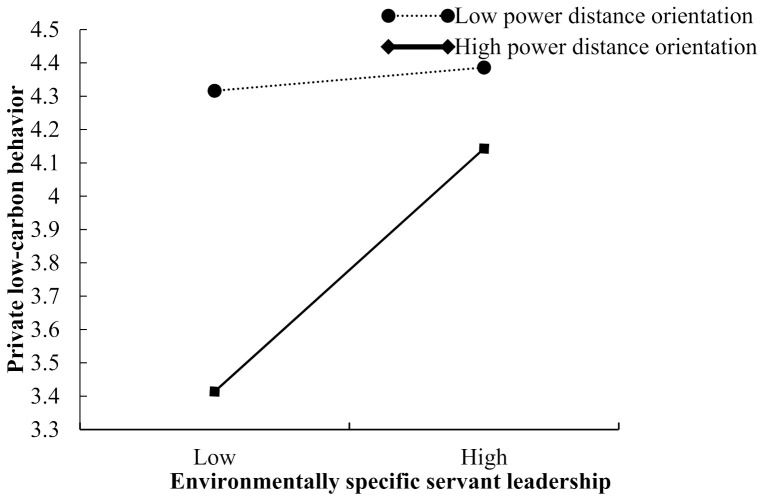
The moderating effect of power distance orientation on the relationship between environmentally specific servant leadership and private low-carbon behavior.

**Figure 4:**
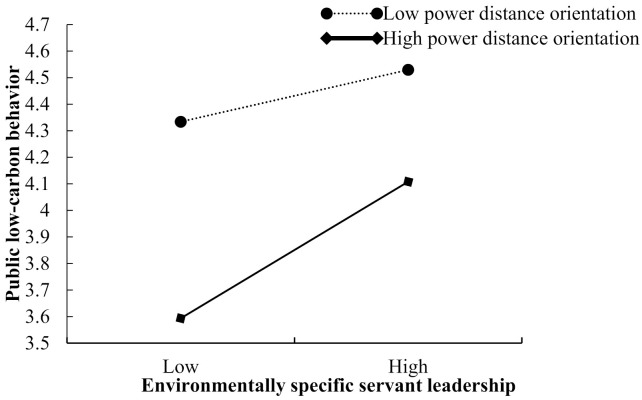
The moderating effect of power distance orientation on the relationship between environmentally specific servant leadership and public low-carbon behavior.

Moreover, the interaction between ESS leadership and power distance orientation is positively associated with environmental self-accountability (β = 0.082, 95% CI = [0.011, 0.153]), private low-carbon behavior (β = 0.186, 95% CI = [0.105, 0.267]), and public low-carbon behavior (β = 0.087, 95% CI = [0.005, 0.168]). Figure 2, Figure 3 and Figure 4 are the simple slopes for different levels of power distance orientation. Thus, Hypotheses 3 and 4 are supported.

Furthermore, the results in Table 3 suggest that power distance orientation moderates the indirect effects of ESS leadership on private low-carbon behavior via environmental self-accountability. That is, environmental self-accountability has a stronger mediation effect on the relationship between ESS leadership and private low-carbon behavior when subordinates have high power distance orientation (i.e., conditional mediation effect = 0.109, 95% CI = [0.066, 0.152]) versus low (i.e., conditional mediation effect = 0.059, 95% CI = [0.019, 0.099]), and the difference between the two indirect effects was 0.050 (95% CI = [0.004, 0.096]). However, although environmental self-accountability has a stronger mediation effect on the relationship between ESS leadership and public low-carbon behavior when subordinates have high power distance orientation (i.e., conditional mediation effect = 0.068, 95% CI = [0.030, 0.106]) versus low (i.e., conditional mediation effect = 0.037, 95% CI = [0.008, 0.065]), the difference between the two indirect effects was not significant (i.e., the difference = 0.031 (95% CI = [0.000, 0.063]). Thus, Hypothesis 5a is supported, but Hypothesis 5b is not supported.

## 5. Discussion

This research constructed a moderated mediation model based on social learning theory to explain the impact of ESS leadership on employees’ low-carbon behavior. Specifically, we examined the impact of ESS leadership on employees’ low-carbon behavior through environmental self-accountability. We used the questionnaire to collect data, and the results of the data analysis ultimately confirmed most of our initial hypothesis. The results indicate that ESS leadership positively influences employees’ low-carbon behavior, and environmental self-accountability mediates the effect of ESS leadership on employees’ low-carbon behavior. This finding validates previous research on pro-environmental behavior, suggesting that ESS leadership promotes pro-environmental behavior and green behavior among employees [10,32,66,67]. This finding also confirms Zheng et al.’s [68] research conclusion: high environmental responsibility positively impacts employees’ environmentally friendly behavior in the workplace. In addition, we found that power distance orientation enhances the impact of ESS leadership on environmental self-accountability and further strengthens the indirect effect of ESS leadership on private low-carbon behavior through environmental self-accountability. This finding validates the previous literature perspective that high power distance orientation enhances the effect of managers’ leadership on employees’ behavior [69,70,71].

However, the results suggest that power distance orientation fails to moderate the indirect effects of ESS leadership on public low-carbon behavior via environmental self-accountability. This result indicates that although subordinates with high power distance orientation tend to follow the values of ESS leadership, they are more likely to conduct low-carbon behavior privately, rather than spend time and energy persuading friends and family members to engage in low-carbon behavior.

### 5.1. Theoretical Contributions

This research has several theoretical implications. First, we constructed a research model to confirm the positive effect of ESS leadership on employees’ low-carbon behavior, contributing to low-carbon behavior literature. Previous studies indicated that environmental psychological factors [13,64,72,73] and demographic factors [74,75], such as low-carbon awareness, low-carbon knowledge, low-carbon intention, gender, marital status, and age, are closely related to low-carbon behavior. However, there is a gap in the low-carbon behavior literature related to the relationship between ESS leadership and employees’ low-carbon behavior in the workplace. This research bridged the gap between ESS leadership and low-carbon behavior by responding to the call of Robertson and Barling [76]. In addition, this research responds to Neo et al. [72] by studying how ESS leadership in the workplace affects employees’ low-carbon behavior. Therefore, this research enriches the literature on low-carbon behavior by shedding light on the relationship between ESS leadership and the low-carbon behavior of employees.

Second, this research unpacks the “black box” underpinning the relationship between ESS leadership and low-carbon behavior by examining the mediating role of environmental self-responsibility. Prior research pointed out that individuals’ values and beliefs towards environmental issues predict low-carbon behavior (for review, see [77]). However, very few studies have examined the psychological process of how leadership shapes low-carbon behavior. Contrary to existing research, we found that environmental self-responsibility is a crucial mechanism underlying the relationship between ESS leadership and employees’ low-carbon behavior. Our finding responds to the study of Wood et al. [78] and further verifies the indispensable role of employees’ environmental self-responsibility in promoting low-carbon behaviors. This research explains why some employees have more low-carbon behaviors and thus provides a more comprehensive understanding of how ESS leadership promotes low-carbon behaviors among employees by looking at the process from perception to behavior.

Third, this research further investigates the question of under what conditions ESS leadership has a stronger effect on employees’ low-carbon behavior. There is almost no existing literature that focuses on the boundary conditions regarding the influencing factors of employees’ low-carbon behavior. To fill this gap, this research tests the moderating effect of power distance orientation on the relationship between ESS leadership and employees’ low-carbon behavior. The results show that compared to employees with low power distance orientation, employees with high power distance orientation learn the environmental protection behavior of ESS leadership more actively and participate in more low-carbon behavior. Moreover, employees with high power distance orientation also agree more with the environmental values of ESS leadership. This results in stronger environmental self-accounting, which is an essential factor to promote low-carbon behavior. Therefore, this finding provides theoretical evidence for enhancing the role of ESS leadership and how to promote low-carbon behavior by employees. At the same time, this research provides a response to Zhang et al. [69] and further verified the notion of a leadership enhancer [22,63].

### 5.2. Practical Implications

This research has some practical implications for managers and organizations. First, climate change and environmental pollution caused by the continuous increase of carbon emissions threaten human survival [4]. For the natural environment, industrial development leads to increased carbon emissions. However, the impact of personal daily behavior on carbon emissions cannot be ignored. It is a critical time to advocate for individuals to increase low-carbon behaviors in their daily lives. Our findings suggest that ESS leadership is positively related to low-carbon behavior among employees. This positive impact helps reduce carbon emissions and achieve the goal of “carbon neutrality”. Managers are encouraged to foster the ESS leadership style in organizations and actively guide employees to engage in low-carbon behavior [79]. For example, leaders should be trained to establish low-carbon values and develop the ability to reduce carbon emissions at work. Additionally, training employees to reduce carbon emissions is also significant. Moreover, managers could provide more resources and improve care for employees who excel at low-carbon behavior to encourage other employees to follow suit [78]. This may be a win–win strategy for employees and organizations. Specifically, employees could obtain resources and organizations could improve green competitive advantage and performance.

Second, the results show that ESS leadership could enhance employees’ low-carbon behavior by improving their environmental self-accountability. This finding suggests that to maximize the effectiveness of ESS leadership, managers and organizations should pay attention to the cultivation of employees’ environmental self-accountability. In response to this, our research provides several recommendations to managers. First and foremost, given that employees tend to imitate the behaviors of leaders, leaders are encouraged to set a good example in caring for the environment. By doing so, employees are predicted to increase their environmental self-accountability [78]. Second, leaders should strive to advocate low-carbon behavior within the organization and nurture a culture of green development, so as to make carbon emissions reduction a consensus of all employees. For example, managers could promote more environmental messages within the organization to attract employees’ attention or to organize employees to participate in environmental charity events to develop their interest. Third, managers could organize training programs related to environmental protection to convey the importance of low-carbon behavior and improve employees’ abilities [41]. It is helpful to enhance employees’ environmental self-accountability and increase low-carbon behavior by raising employees’ awareness of the importance of environmental protection.

Third, various industries, especially energy-intensive and carbon-intensive industries, should strive to reduce carbon emissions. After the Chinese government pledged to achieve peak carbon emissions by 2030 and carbon neutrality by 2060, reducing carbon emissions has become an important development strategy for China’s industry. Since leaders have an irreplaceable influence on employees’ behaviors and the development of companies, leaders must have a clear understanding of the development of the industry. Only in this way can they better lead the development of enterprises. Here are three suggestions. First, leaders should be aware of the importance of reducing carbon emissions for the development of the industry and set the reduction of carbon emissions as one of the main development goals of the enterprise, so as to lead the low-carbon development of the industry. Second, leaders should clarify the carbon emission requirements of policies and regulations on the industry, and strive to learn and apply low-carbon technologies in enterprise development to reduce carbon emissions and meet the requirements [80]. Third, leaders should actively participate in industry conferences and accumulate experience in reducing carbon emissions, so as to contribute to the low-carbon development of the industry.

### 5.3. Limitations and Future Research

This research may also have several limitations. First, while the current research examines the impact of ESS leadership on employees’ low-carbon behavior with multi-wave data, our research design is cross-sectional, which limits our inference of causality. Future research is encouraged to explore whether ESS leadership always has a positive impact on employees’ low-carbon behavior by utilizing a longitudinal design. Moreover, this research was conducted only in Shandong Province, China, which limits the generalizability of the results to some degree. Future research could examine whether ESS leadership influences employees’ low-carbon behavior through environmental self-accountability in other provinces and countries, particularly in developed countries with completely different social cultures from China. 

Second, this research only focuses on ESS leadership and ignores other organization members’ impact on employees’ low-carbon behavior. The relationship between team members is closer. The words and deeds of coworkers may also have a potentially significant impact on focal employees’ low-carbon behavior and awareness. Future research could investigate the influence of lifestyle habits, low-carbon values, and behaviors of other organization members (e.g., coworkers or team members) on focal employees’ environmental self-accountability and low-carbon behavior.

Third, our research only investigates the moderating role of power distance orientation. However, aside from power distance orientation, other factors, such as organization internal competition (e.g., peer pressure), individual consciousness (e.g., low-carbon awareness, low-carbon intention), corporate culture, and government policy requirements might also influence employees’ low-carbon behavior. For example, in terms of organization internal competition, employees who consciously perform well in low-carbon behavior may receive additional rewards from the organization (e.g., salary, promotion). Such experience may lead coworkers to exhibit similar behaviors. Therefore, we encourage future research to explore the boundary effect of these factors.

## 6. Conclusions

Following the primary tenet of social learning theory, this research expands our understanding of why, how, and when ESS leadership affects employees’ low-carbon behavior by investigating practically meaningful mediator and boundary conditions of ESS leadership. We found that ESS leadership positively impacts employees’ low-carbon behavior, including private low-carbon behavior and public low-carbon behavior. Moreover, environmental self-accountability plays a mediating role between ESS leadership and employees’ low-carbon behavior. In other words, ESS leadership improves employees’ low-carbon behavior by driving their environmental self-accountability. In addition, we also found that power distance orientation strengthens the positive effect of ESS leadership on environmental self-accountability, low-carbon behavior, as well as the indirect effect of ESS leadership on private low-carbon behavior. Our findings contribute to the literature by investigating the antecedent variable of low-carbon behavior, its underlying mechanism, and potential contextual factors. Meanwhile, our research provides managerial implications for organizations to help achieve reduced carbon and carbon neutrality. We encourage future studies performing more profound research based on our results, such as expanding the scope of data collection and testing different underlying mechanisms and contextual factors.

## Figures and Tables

**Table 1:** Confirmatory factor analysis.

Model	χ^2^	df	χ^2^/df	CFI	TLI	RMSEA	SRMR
Six-factor model: ESTL, PDO, ESSL, ESA, PrLCB, PuLCB	952.287	804	1.184	0.983	0.981	0.020	0.035
Five-factor model: ESTL, PDO, ESSL, ESA, PrLCB + PuLCB	1269.251	809	1.569	0.946	0.942	0.034	0.043
Four-factor model: ESTL, PDO, ESSL, ESA + PrLCB + PuLCB	1510.117	813	1.857	0.918	0.913	0.042	0.048
Three-factor model: ESTL, ESSL, PDO + ESA + PrLCB + PuLCB	2308.742	816	2.829	0.824	0.815	0.062	0.081
Two-factor model: ESTL, ESSL + PDO + ESA + PrLCB + PuLCB	3563.175	818	4.356	0.677	0.660	0.083	0.107
One-factor model: ESTL + ESSL + PDO + ESA + PrLCB + PuLCB	5596.765	819	6.834	0.438	0.409	0.110	0.138

Note: N = 483. ESTL = environmentally specific transformational leadership; PDO = power distance orientation; ESSL = environmentally specific servant leadership; ESA = environmental self-accountability; PrLCB = private low-carbon behavior; PuLCB = public low-carbon behavior.

**Table 2:** Means, standard deviations, and correlations.

	Mean	SD	1	2	3	4	5	6	7	8	9
1. Age	30.99	7.27	-								
2. Gender	0.40	0.49	−0.072	-							
3. Education level	2.09	0.93	0.189 **	0.123 **	-						
4. ESTL	4.74	0.86	−0.027	−0.003	−0.065	**(0.922)**					
5. PDO	3.79	0.92	−0.070	−0.010	−0.069	−0.098 *	**(0.859)**				
6. ESSL	4.85	0.85	−0.118 **	−0.007	−0.048	0.283 **	−0.041	**(0.911)**			
7. ESA	4.97	0.74	−0.051	−0.008	0.058	0.227 **	−0.159 **	0.331 **	**(0.754)**		
8. PrLCB	4.62	0.91	−0.061	0.038	0.034	0.233 **	−0.306 **	0.299 **	0.391 **	**(0.754)**	
9. PuLCB	4.74	0.87	−0.070	−0.067	−0.088	0.303 **	−0.336 **	0.296 **	0.311 **	0.393 **	**(0.806)**

Note: N = 483. Internal consistent reliability (alpha) coefficients are shown along the diagonal in bold. Gender, 0 = male; 1 = female. Education level, 1 = high school or below, 2 = associate degree, 3 = bachelor’s degree or above. ** *p* < 0.01, * *p* < 0.05, same for following tables.

**Table 3:** Regression results for directing, mediating, and moderating effects.

Predictor	Effect	S.E.	95% CI	Significance
M: Environmental self-accountability				
X: ESS leadership	0.274	0.036	[0.202, 0.345]	<0.001
W: Power distance orientation	−0.129	0.036	[−0.198, −0.059]	<0.001
Interaction: X × W	0.082	0.036	[0.011, 0.153]	<0.050
Y1: Private low-carbon behavior				
X: ESS leadership	0.163	0.035	[0.095, 0.232]	<0.001
M: Environmental self-accountability	0.307	0.050	[0.209, 0.405]	<0.001
W: Power distance orientation	−0.274	0.039	[−0.350, −0.198]	<0.001
Interaction: X × W	0.186	0.041	[0.105, 0.267]	<0.001
Y2: Public low-carbon behavior				
X: ESS leadership	0.163	0.035	[0.095, 0.232]	<0.001
M: Environmental self-accountability	0.191	0.049	[0.094, 0.288]	<0.001
W: Power distance orientation	−0.292	0.039	[−0.367, −0.216]	<0.001
Interaction: X × W	0.087	0.042	[0.005, 0.168]	<0.050
Indirect effect of X on Y1 via M				
M: Environmental self-accountability	0.084	0.018	[0.049, 0.119]	<0.001
Conditional indirect effect(s) at values of power distance orientation (X → M → Y1)				
W: Power distance orientation				
−1 SD	0.059	0.020	[0.019, 0.099]	<0.010
+1 SD	0.109	0.022	[0.066, 0.152]	<0.001
Difference	0.050	0.023	[0.004, 0.096]	<0.050
Indirect effect of X on Y2 via M				
M: Environmental self-accountability	0.052	0.015	[0.023, 0.082]	<0.001
Conditional indirect effect(s) at values of power distance orientation (X → M → Y2)				
W: Power distance orientation				
−1 SD	0.037	0.015	[0.008, 0.065]	<0.050
+1 SD	0.068	0.019	[0.030, 0.106]	<0.001
Difference	0.031	0.016	[0.000, 0.063]	n.s.

Note. N = 483. S.E. = standard error. CI = confidence interval. Values for quantitative moderators are the mean and plus/minus one SD from mean.

## Data Availability

The data are not publicly available due to privacy and ethical considerations.
